# Benefit from Adjuvant TKIs Versus TKIs Plus Chemotherapy in *EGFR*-Mutant Stage III-pN2 Lung Adenocarcinoma

**DOI:** 10.3390/curroncol28020135

**Published:** 2021-04-07

**Authors:** Qiwen Li, Li Ma, Bo Qiu, Yuzhi Wen, Wenhua Liang, Wanming Hu, Naibin Chen, Tian Zhang, Shuangbing Xu, Lingjuan Chen, Minzhang Guo, Yi Zhao, Songran Liu, Jinyu Guo, Junye Wang, Siyu Wang, Xin Wang, Qingsong Pang, Hao Long, Hui Liu

**Affiliations:** 1 State Key Laboratory of Oncology in South China, Department of Radiation Oncology, Collaborative Innovation Center for Cancer Medicine, Sun Yat-sen University Cancer Center, Guangzhou 510060, China; liqw@sysucc.org.cn (Q.L.); qiubo@sysucc.org.cn (B.Q.); wenyzh@sysucc.org.cn (Y.W.); chennb@sysucc.org.cn (N.C.); guojingyu@sysucc.org.cn (J.G.); 2 Department of Radiation Oncology, National Cancer Center, Cancer Hospital & Shenzhen Hospital, Chinese Academy of Medical Sciences and Peking Union Medical College, Shenzhen 518116, China; ml_1990@126.com; 3 State Key Laboratory of Respiratory Disease, Department of Thoracic Surgery and Oncology, The First Affiliated Hospital of Guangzhou Medical University, National Clinical Research Center of Respiratory Disease, Guangzhou 510060, China; liangwh1987@163.com (W.L.); guominzhang199106@163.com (M.G.); yizhao0606@163.com (Y.Z.); 4 State Key Laboratory of Oncology in South China, Department of Pathology, Collaborative Innovation Center for Cancer Medicine, Sun Yat-sen University Cancer Center, Guangzhou 510060, China; huwm@sysucc.org.cn (W.H.); liusr@sysucc.org.cn (S.L.); 5 Department of Radiation Oncology, Tianjin Medical University Cancer Institute and Hospital, Tianjin 300060, China; zhangtian8984@tijmu.edu.cn (T.Z.); pangqingsong@tjmuch.com (Q.P.); 6 Union Hospital Cancer Center, Tongji Medical College, Huazhong University of Science and Technology, Wuhan 430000, China; xsb723@hust.edu.cn (S.X.); chenlingjuan@hust.edu.cn (L.C.); 7 State Key Laboratory of Oncology in South China, Department of Thoracic Surgery, Collaborative Innovation Center for Cancer Medicine, Sun Yat-sen University Cancer Center, Guangzhou 510060, China; wangjy@sysucc.org.cn (J.W.); wangsy@sysucc.org.cn (S.W.); wangxin@sysucc.org.cn (X.W.); longhao@sysucc.org.cn (H.L.)

**Keywords:** lung adenocarcinoma, N2, *EGFR* mutation, adjuvant TKIs, chemotherapy

## Abstract

Background: Recent studies have demonstrated benefits from adjuvant tyrosine-kinase inhibitors (TKIs) compared with chemotherapy in non-small cell lung cancer. We launched a multi-center retrospective study to evaluate the efficacy and toxicity of adjuvant TKIs with or without chemotherapy in epidermal growth factor receptor (*EGFR*)-mutant stage III-pN2 lung adenocarcinoma. Methods: Two hundred and seventy-four consecutive cases with stage III-pN2 lung adenocarcinoma and complete resection have been investigated. Clinic-pathologic characteristics, adjuvant treatments, long-term survivals, and toxicities were documented. Risk factors of distant metastasis-free survival (DMFS), disease-free survival (DFS), and overall survival (OS) were evaluated. Results: There were 52 (19.0%) patients treated with adjuvant TKIs alone, 199 (72.6%) with adjuvant chemotherapy alone, and 23 (8.4%) with both. After a median follow-up time of 29 months, the two-year DMFS, DFS, and OS was 61.2%, 54.1%, and 91.2%, respectively. According to univariable analyses, the risk factors were lymphovascular invasion (*p* < 0.001), extranodal extension (*p* = 0.005), and adjuvant systemic therapy (*p* = 0.006) for DMFS, *EGFR* mutation type (*p* = 0.025), lymphovascular invasion (*p* = 0.013), extranodal extension (*p* = 0.004), and adjuvant systemic therapy (*p* < 0.001) for DFS, and *EGFR* mutation type (*p* < 0.001) for OS. Multivariable analyses indicated that the independent prognostic factors were adjuvant systemic therapy (TKIs vs. TKIs+chemotherapy, Harzard ratio (HR) = 0.40; *p* = 0.036; TKIs vs. chemotherapy, HR = 0.38; *p* = 0.004), lymphovascular invasion (yes vs. no, HR = 2.22; *p* = 0.001) for DMFS, and adjuvant systemic therapy (TKIs vs. TKIs+chemotherapy, HR = 0.42; *p* = 0.034; TKIs vs. chemotherapy, HR = 0.33; *p* < 0.001) for DFS. No significant difference was found in the incidence of Grade 3–4 toxicities between groups (*p* = 0.445). Conclusions: Adjuvant TKIs might be a beneficial choice compared with adjuvant chemotherapy or combination systemic treatments.

## 1. Background

Roughly 40–50% of lung adenocarcinomas diagnosed in China harbor the mutant epidermal growth factor receptor (*EGFR*) gene [1]. Although early-stage lung cancer is treated surgically with curative intent, recurrence rates after complete anatomic resection remain unacceptably high, ranging from 30% to 80% [2,3,4]. The two-year survival for patients with stage III disease is less than 50% despite definitive therapy [5]. The Lung Adjuvant Cisplatin Evaluation (LACE), a pooled analysis of five large trials (4584 patients), demonstrated a five-year overall survival (OS) benefit of 5.4% with adjuvant chemotherapy [6]. This is a fairly modest gain considering the toxicity associated with cisplatin-based chemotherapy and leaves us in dire need of novel adjuvant approaches to improve cure rates. The unprecedented success of small-molecule *EGFR* tyrosine kinase inhibitors (TKIs) challenged the standard of care in the adjuvant setting. In the National Cancer Institute of Canada (NCIC) phase 3 BR.19 trial, patients with stage IB-IIIA non-small cell lung cancer (NSCLC) were randomized, following surgical resection and optional adjuvant chemotherapy, to either two years of adjuvant gefitinib or placebo. Unfortunately, BR.19 was underpowered, terminated early, and nonenriched for the relevant population; thus, no statistically robust conclusions was drawn [7]. RADIANT was a randomized, double-blind, controlled phase 3 study evaluating whether adjuvant erlotinib could improve survival in completely resected stage IB to IIIA NSCLC when positive *EGFR* by immunohistochemistry or *EGFR* amplification by fluorescence in situ hybridization was identified. Although neither disease-free survival (DFS) nor OS was statistically improved, a subset analysis of patients with deletion exon 19 or exon 21 L858R showed a remarkable advantage in DFS (Harzard ratio (HR) 0.61; *p* = 0.04), implying the importance of proper biomarker selection in future studies [8]. Recently, the randomized, open-label, phase 3 ADJUVANT/CTONG1104 study has suggested a significantly improved DFS in stage II-IIIA patients with *EGFR* exon 19 or 21 mutation who were treated with adjuvant gefitinib for up to 24 months, compared with those with adjuvant vinorelbine plus cisplatin [9]. Another randomized, phase 2 trial also demonstrated improved two-year DFS and better tolerability in patients with *EGFR*-mutant stage IIIA NSCLC after adjuvant erlotinib compared with chemotherapy [10]. The latest published results of the phase 3 ADAURA trial has provided evidence for a prolonged DFS in stage IB-IIIA *EGFR*-mutant NSCLC, where osimertinib was compared with placebo [11]. As a result, the TKI monotherapy is becoming an option in the adjuvant treatment of NSCLC with *EGFR* mutation.

Whether adjuvant chemotherapy and concurrent/subsequent TKIs brings extra benefit remained unclear. There were several phase 2/3 studies performed in advanced NSCLC, but no consistent conclusion has been achieved. The INTACT and TRIBUTE study revealed no significant difference in OS or progression-free survival (PFS) between combination therapy and TKIs alone as first-line treatment in newly diagnosed, advanced NSCLC [12,13,14]. The results of Wen et al. showed significantly improved PFS following combination therapy in *EGFR*-mutant advanced NSCLC, while no OS benefit was reported [15]. However, The Japanese phase 2 study-NEJ005 revealed both superior OS and PFS of the combination therapy of TKIs and chemotherapy [16].

Therefore, we performed this multi-center retrospective study in R0-resected *EGFR*-mutant pathologic N2 lung adenocarcinoma to evaluate the optimal adjuvant systemic treatments and other prognostic factors of clinical outcomes.

## 2. Methods

### 2.1. Study Population

The study was conducted in accordance with ethical standards of the Helsinki Declaration and the Ethics Committee of Sun Yat-sen University Cancer Center (YB2017-047). Because it was a retrospective and anonymous study, a waiver of authorization was required and granted.

The study was conducted at tertiary medical centers in China, including the Sun Yat-sen University Cancer Center, the Tianjin Medical University Cancer Institute and Hospital, the First Affiliated Hospital of Guangzhou Medical University, and the Union Hospital Cancer Center, Tongji Medical College. Consecutive patients operated for lung cancer and tested for *EGFR* mutation with surgical specimen during the period September 2001 to December 2016 were retrospectively screened. All cases of lung adenocarcinoma were confirmed by histology. Full baseline evaluations had to be completed before treatment, including enhanced chest computed tomography (CT), brain magnetic resonance imaging (MRI), bone scan, positron emission tomography/computed tomography (PET)/CT (optional), and fibrotic bronchoscopy and pathologic examination. Clinical and pathologic staging was based on AICC/UICC 7th staging criteria [17]. Finally, patients with R0 resected stage III-pN2 *EGFR*-mutant lung adenocarcinoma were included in the analysis, but excluded if they were lost to follow-up within six months after surgery.

### 2.2. N Sub-Staging

N2 nodal status was carefully reviewed from pre-treatment work-ups and postoperative pathologic report, and was further divided into four groups: unforeseen N2 (IIIA1-2), minimal N2/single station at staging (IIIA3), and bulky and/or multilevel N2 at staging (IIIA4), according to the Robinson classification [18]. The IIIA1 disease was not separated from IIIA2 because intraoperative mediastinal lymph node pathologic staging was not routinely performed in these medical centers.

### 2.3. EGFR Genotyping

Paraffin-embedded, formalin-fixed specimens obtained from surgical tissues were prepared for the extraction of genomic DNA. Predominant *EGFR* mutations were tested via the amplification-refractory mutation system (ARMS), including point mutation in exon 21 and short in-frame deletions in exon 19.

### 2.4. Pathologic Examination

Slides with hematoxylin-eosin staining, immune-histochemical staining, and elastic staining were carefully reviewed by experienced pathologists from the four medical centers, based on the same standard. Complete resection was defined as microscopically proven free resection margins. The presence of malignant cells extension into perinodal adipose tissue through the nodal capsule was considered as extranodal extension. Tumor cells found in lymphatic lumen, vascular lumen, or the space around nerves was defined as lymphatic invasion, vascular invasion, or perineural invasion.

### 2.5. Treatments

Before surgery, all cases with multilevel or/and bulky N2 were discussed by a multidisciplinary team (MDT). The patients went to surgery unless the tumor was regarded as resectable. The surgical procedure, lobectomy, pneumonectomy, or sublobectomy was decided by individual surgeons according to the size and location of the disease, as well as the patients’ pulmonary and cardiac function. Station 1 nodes were routinely dissected. Ipsilateral mediastinal lymphotomy was performed, usually including dissection of station 2R, 4R, and 7–9 for right lung cancer and 4L, 5, 6, and 7–9 for left lung cancer.

Adjuvant systemic therapies included TKIs alone (two months at least), chemotherapy alone (at least four cycles of platinum based two-drug regimen), and the combination of TKIs and chemotherapy. Either of the following regimens was administered as adjuvant chemotherapy: paclitaxel+carboplatin, pemetrexed+cisplatin/carboplatin, docetaxel+cisplatin/nedaplatin, or vinorelbine detartrate+cisplatin. Either gefitinib, erotinib afatinib, or icotinib was administered as adjuvant TKIs.

### 2.6. Follow-Up

Chest and upper abdominal CT and brain MRI were performed one to two months after surgery, every three to six months in the following two years, and every six to twelve months thereafter. Bone scan, PET/CT, and biopsy were suggested by a physician if necessary. DFS or OS was defined as the time from surgery to death/first recorded treatment failure, or to death from any cause, respectively. Both local and distant relapses were documented. Local recurrence was defined as any recurrence found at the surgical margin, ipsilateral hemi-thorax (except for multiple lesions in the ipsilateral lung), or regional lymph nodes. Any relapse occurring elsewhere was regarded as distant metastasis. Distant metastasis-free survival (DMFS) was defined as the time from surgery to first distant metastasis. Treatment-related toxicities were assessed from the start of adjuvant treatment to two months from the end of treatment. Common Terminology Criteria for Adverse Events (CTCAE) v4.0 was used to evaluate the toxicities.

### 2.7. Statistical Methods

Survival was evaluated by the Kaplan–Meier method and compared in univariable analysis using the log-rank test. Factors with *p* < 0.1 were included in the Cox proportional hazards model using enter procedure and assessed in multivariable analyses; *p*-values < 0.05 (two-sided) were regarded as statistically significant. Missing data was excluded from the analysis. All tests were conducted using SPSS 22.0 (SPSS Inc., Chicago, IL, USA).

## 3. Results

**Figure 1:**
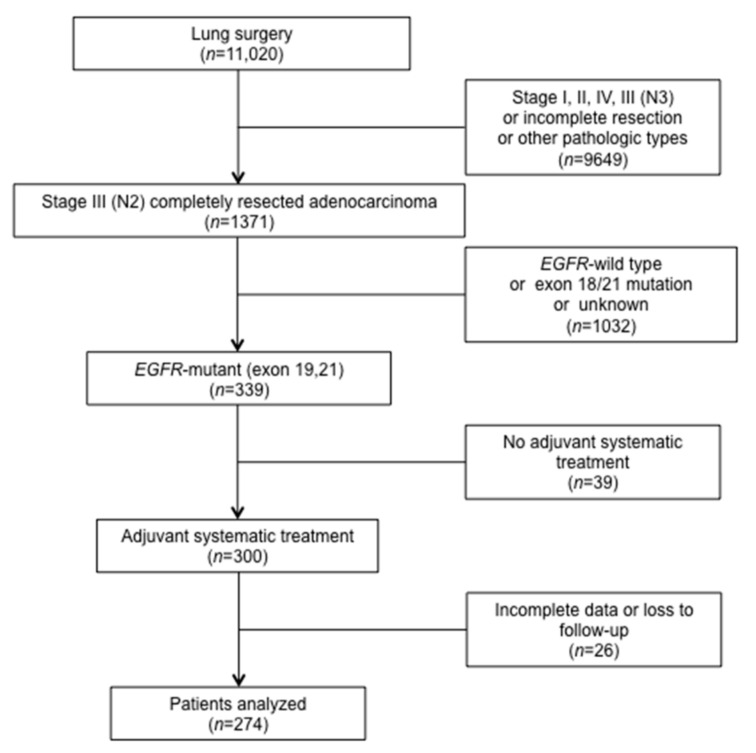
Flowchart for patient enrollment. *EGFR*: epidermal growth factor receptor.

In the screening of 11,020 consecutive patients registered in the institutional databases, a total of 274 patients met the study criteria and were included in the analysis (Figure 1). PET/CT results were available in 93 (34%) patients. Table 1 details the clinic-pathologic characteristics and treatment-related parameters.

There were 199 (72.6%) patients treated with adjuvant chemotherapy alone (median number of cycles, 4; range, 4–6), 52 (19.0%) with TKIs alone (median duration, 11 months; range, 3–28 months) and 23 (8.4%) with both (median number of chemotherapy cycles, 4; range, 4–6; median TKI duration, 13 months; range, 4–18 months). Of those who had adjuvant TKIs, only 20 were included in a clinical trial (NCT01683174) and 32 refused or felt intolerant to adjuvant chemotherapy. All of the 23 patients treated with combination therapy started TKIs after the completion of chemotherapy based on individual decisions made by the patients and physicians. The median gap between surgery and adjuvant chemotherapy was 24 (range, 5–91) days and 101 (range, 8–251) days between surgery and adjuvant TKIs. The detailed regimens of adjuvant systemic therapies are presented in Table 2.

### 3.1. Patterns of Recurrence

Median follow-up time was 29 (range, 6–133) months. One hundred and forty-nine (54.4%) patients relapsed, including 16 (5.8%) with local recurrence only, 84 (30.7%) with distant metastases only, 33 (12.0%) with both as first recurrence sites, and 16 (5.8%) with unknown sites.

After the first failure, 14 patients had palliative therapy because of economic reasons or personal decisions. Nine patients did not receive further treatment. The other 126 patients received salvage treatments, which are detailed in Supplementary Table S1.

### 3.2. Distant Metastasis and Prognostic Factors

**Figure 2:**
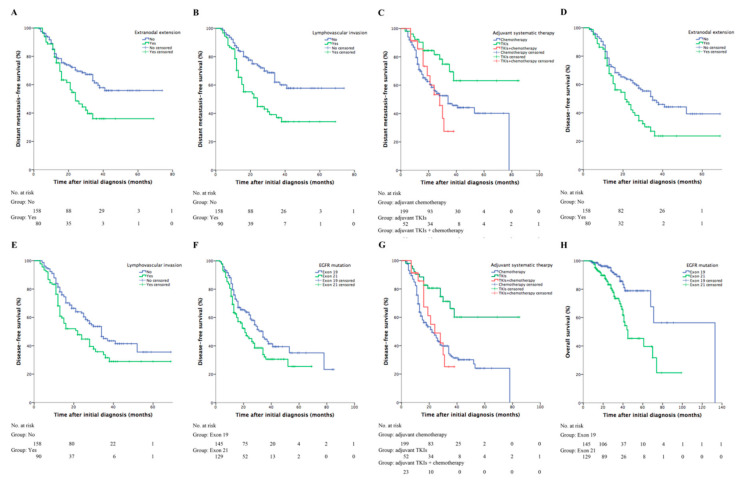
Clinical outcomes based on different prognostic factors. The DMFS curves for patients sub-grouped by (**A**) pathologic extranodal extension (two-year DMFS, yes vs. no, 48.5% vs. 70.4%, *p* = 0.005), (**B**) lymphovascular invasion (44.8% vs. 74.0%, yes vs. no, *p* < 0.001), and (**C**) system adjuvant therapy (adjuvant TKIs vs. adjuvant TKIs+chemotherapy vs. adjuvant chemotherapy, 81.5% vs. 53.4% vs. 56.4%, *p* = 0.006). The DFS curves for patients sub-grouped by (**D**) pathologic extranodal extension (two-year DFS, yes vs. no, 42.1% vs. 62.9%, *p* = 0.004), (**E**) lympovascular invasion (yes vs. no, 44.7% vs. 63.0%, *p* = 0.013), (**F**) *EGFR* mutations (exon 19 vs. 21, 61.8% vs. 45.2%, *p* = 0.025), and (**G**) systemic adjuvant therapy (adjuvant TKIs vs. adjuvant TKIs+chemotherapy vs. adjuvant chemotherapy, 80.6% vs. 48.9% vs. 47.4%, *p* < 0.001). (**H**) The OS curve for patients with *EGFR* exon 19 or 21 mutation (two-year OS, exon 19 vs. 21, 96.2% vs. 85.5%, *p* < 0.001). DMFS: distant metastasis-free survival; TKIs: tyrosine-kinase inhibitors; DFS: disease-free survival; *EGFR*: epidermal growth factor receptor; OS: overall survival.

The two-year distant metastasis-free survival (DMFS) was 61.2% (95% CI, 54.9–67.5%). In univariable analysis, there was a tendency towards distant failure in patients with pathologic extranodal extension (two-year DMFS, 48.5% vs. 70.4%, *p* = 0.005, Figure 2A) or lymphovascular invasion (44.8% vs. 74.0%, *p* < 0.001, Figure 2B). Patients receiving adjuvant TKIs alone showed a higher two-year DMFS compared with other adjuvant therapies (TKIs vs. TKIs+chemotherapy vs. chemotherapy, 81.5% vs. 53.4% vs. 56.4%, *p* = 0.006, Figure 2C). Multivariable analysis addressed lymphovascular invasion (HR = 2.22; 95% CI, 1.38–3.57; *p* = 0.001) and adjuvant systemic treatments (TKIs vs. TKIs+chemotherapy, HR = 0.40; 95% CI, 0.17–0.94; *p* = 0.036; TKIs vs. chemotherapy, HR = 0.38; 95% CI, 0.20–0.73; *p* = 0.004) as independent prognostic factors of DMFS.

### 3.3. Survival and Prognostic Factors

At the last follow-up, there were 59 deaths recorded. All deaths were caused by disease progression.

The estimated two- and three-year DFS was 54.1% (95% CI, 47.8–60.4%) and 38.1% (95% CI, 31.2–45.0%), respectively. The estimated median DFS was 28 (range, 23–33) months. Prognostic factors on DFS revealed by univariable analysis included extranodal extension (two-year DFS, yes vs. no, 42.1% vs. 62.9%, *p* = 0.004, Figure 2D), lympovascular invasion (yes vs. no, 44.7% vs. 63.0%, *p* = 0.013, Figure 2E), *EGFR* mutation type (exon 19 vs. 21, 61.8% vs. 45.2%, *p* = 0.025, Figure 2F), and adjuvant treatments (TKIs vs. TKIs+chemotherapy vs. chemotherapy, 80.6% vs. 48.9% vs. 47.4%, *p* < 0.001, Figure 2G). Adjuvant systemic treatments (TKIs vs. TKIs+chemotherapy, HR = 0.42; 95% CI, 0.19–0.94; *p* = 0.034; TKIs vs. chemotherapy, HR = 0.33; 95% CI, 0.18–0.61; *p* < 0.001) were identified as being statistically predictive of DFS in multivariable analysis.

The estimated two- and three-year OS was 91.2% (95% CI, 87.5–94.9%) and 80.9% (95% CI, 75.0–86.8%), respectively. The estimated median OS was 70 (range, 60–80) months. *EGFR* mutation type was significantly associated with two-year OS (exon 19 vs. 21, 96.2% vs. 85.5%, *p* < 0.001, Figure 2H). Salvage TKIs was not a prognostic factor of OS (*p* = 0.52).

Table 3 summarizes the results of univariable and multivariable analyses on all clinical outcomes.

### 3.4. Additional Analysis

To overcome potential calendar time bias, 73 patients who received surgery from 2015 to the present have been extracted for further analysis. The characteristics are shown in Supplementary Table S2.

The two-year DMFS was 49.1% (95% CI, 34.8–63.4%). In the univariable analysis, *EGFR* mutation type (two-year DMFS, exon 19 vs. 21, 65.0% vs. 28.9%, *p* = 0.046), lymphovascular invasion (yes vs. no, 37.2% vs. 64.5%, *p* = 0.018), adjuvant systemic therapy (TKIs vs. TKIs+chemotherapy vs. chemotherapy, 80.4% vs. 67.7% vs. 27.0%, *p* = 0.004), and postoperative radiotherapy (PORT vs. no PORT, 0% vs. 52.1%, *p* < 0.001) were predictive of distant failure. Multivariable analysis indicated that lymphovascular invasion (HR = 3.66; 95% CI, 9.23–1.45; *p* = 0.006), adjuvant systemic treatments (TKIs vs. TKIs+chemotherapy, HR = 0.36; 95% CI, 0.085–1.52; *p* = 0.165; TKIs vs. chemotherapy, HR = 0.19; 95% CI, 0.054–0.67; *p* = 0.010), and PORT (HR = 10.1; 95% CI, 40.04–2.55; *p* < 0.001) were independent risk factors of DMFS.

The estimated two- and three-year DFS was 42.7% (95% CI, 29.0–56.4%) and 26.6% (95% CI, 11.3–41.9%), respectively. Prognostic factors on DFS revealed by univariable analysis included *EGFR* mutation type (exon 19 vs. 21, 57.7% vs. 45.3%, *p* = 0.045), postoperative radiotherapy (PORT vs. no PORT, 0% vs. 22.5%, *p* < 0.001,), and adjuvant treatments (TKIs vs. TKIs+chemotherapy vs. chemotherapy, 75.0% vs. 54.2% vs. 21.0%, *p* = 0.003). Adjuvant systemic treatments (TKIs vs. TKIs+chemotherapy, HR = 0.50; 95% CI, 0.14–1.77; *p* = 0.282; TKIs vs. chemotherapy, HR = 0.26; 95% CI, 0.087–0.78; *p* = 0.016) and postoperative radiotherapy (PORT vs. no PORT, HR = 6.37; 95% CI, 20.61–1.97; *p* = 0.002) were predictive of DFS in multivariable analysis.

Supplementary Table S3 summarizes the results of univariable and multivariable analyses on clinical outcomes in this subgroup.

### 3.5. Toxicities

The overall incidence of Grade 3–4 toxicities of patients treated with chemotherapy, TKIs, and combination therapy was 25.1% (50/199), 9.6% (5/52), and 17.4% (4/23), respectively (*p* = 0.445). The most frequently reported Grade 3–4 toxicities was leukopenia (16.6%), rash (3.8%), and leukopenia (17.4%) in each group, respectively. Other details of severe toxicities are presented in Table 4. No treatment-related death was documented.

## 4. Discussion

Stage III-pN2 NSCLC represents a subpopulation with aggressive biological behavior and has the greatest demand for multimodality treatments. In our previous study analyzing the clinical outcomes of completely resected stage III lung adenocarcinoma harboring mutant *EGFR*, more than half of all cases ultimately developed distant metastasis [19]. To investigate the optimal adjuvant systemic treatment, we then combined consecutive patients from four large medical centers in China and launched this retrospective study, comparing the efficacy and toxicity of adjuvant TKIs, chemotherapy, and combination therapy.

The two-year DFS in the current study were 80.6% in the TKI alone group, 48.9% in the combination group, and 47.4% in the chemotherapy alone group. Similarly, the three-year DFS reported in the phase 2 EVAN study were 54.2% and 19.8% in patients treated with erotinib and chemotherapy, respectively [10]. In ADJUVANT/CTONG1104, the three-year DFS was reported as 34% and 27% in those who received gefitinib and chemotherapy [9], and in the AUDURA trial, the three-year DFS reported with or without osimertinib was 90% and 44%, respectively [11]. Our study suggested that both distant control and DFS were significantly improved with the administration of adjuvant TKIs alone, although no statistical difference was found in OS. Overall, the combination strategy did not show any advantage over chemotherapy or TKIs alone. The same trend remained in the analysis of the subgroup treated from 2015 to the present, although the advantage of TKIs over the combination therapy was not statistically significant, probably because of the limited sample size. TKIs alone seemed to be a beneficial choice in the adjuvant setting, with a favorable safety profile as well.

The ADJUVANT/CTONG1104 trial evaluating gefitinib in the adjuvant setting demonstrated increased DFS, but not OS, and reported common recurrence in the central nervous system [9]. A potential role of adjuvant TKIs was suggested but did not lead to changes of clinical practice. Recently, the phase 3 AUDURA trial has come up with an impressive improvement with adjuvant osimertinib in *EGFR*-mutant stage IB-IIIA NSCLC. The benefit favoring osimertinib was observed regardless of the presence of adjuvant chemotherapy. Compared to those with earlier stage, stage IIIA patients had the smallest hazard ratio for disease recurrence or death when receiving osimertinib. Besides, adjuvant osimertinib significantly reduces the chance of central nervous system metastasis [11]. These results have brought several considerations. First, osimertinib, which could induce apoptosis, seemed to be more potent than gefitinib or erlotinib in mutant EGFR. Second, recognizing patients with higher recurrence risk might help to select treatment candidates. For instance, Ni et al. created a prognostic model including immunohistochemical markers, e.g., Ki67, CK20, tumor size, and N stage [20]. Identifying patients with a high risk of central nervous system metastasis might also be beneficial for choosing the adjuvant TKI. Third, it is essential to answer the question of who is more sensitive to adjuvant TKIs. Both the results from the AUDURA trial and a recent meta-analysis suggested that patients with stage IIIA NSCLC might benefit more from adjuvant TKIs than stage I patients after radical surgery [11,21]. The predictive value of biomarkers, including baseline T790M mutation status and others with a plasma-derived circulating tumor DNA EGFR mutation status, are being explored [22].

Evidence from an in vitro study has suggested that combing EGFR-TKIs and either cisplatin or paclitaxel resulted in increased apoptotic response and a synergistic effect on cytotoxicity [23]. Meanwhile, cytotoxic agents might play a role in altering the resistance mechanisms of EGFR-TKIs. In the past decade, four randomized phase III studies failed to demonstrate the superiority of combination therapy compared with TKIs alone in advanced NSCLC populations [12,13,14,24]. The results may partly be due to nonselective enrolled patients because the *EGFR* mutation was not routinely tested at that time. More recently, a retrospective study found that patients with the *EGFR* mutant advanced NSCLC who received the combination therapy of TKIs and chemotherapy could achieve longer PFS than those who receive TKIs alone (median, 20.5 vs. 16 months, *p* = 0.036), but no difference was found in OS (median, 36 vs. 29 months, *p* = 0.19) [15]. Meanwhile, the phase 2 study-NEJ005 reported that patients with the *EGFR* mutant NSCLC could benefit from combined chemotherapy and TKIs with a longer OS compared with TKIs alone (median, 41.9 vs. 30.7 months, *p* = 0.036) [16]. In our study, the clinical outcomes between chemotherapy and combination therapy were similar, with a higher rate of Grade 3–4 toxicities in the combination group. Until now, there is no agreement regarding whether TKIs plus chemotherapy is superior to monotherapy. More high-qualified prospective data is required.

Other attempts have been made to overcome primary resistance to *EGFR*-TKIs in NSCLC. Recently, in patients diagnosed with both advanced NSCLC and type 2 diabetes, when gefitinib and metformin were used simultaneously, extraordinarily favorable DFS and OS were noticed [25]. An ongoing phase 2 study will evaluate the efficacy and safety of gefitinib with metformin as first-line therapy of stage IIIb-IV *EGFR*-mutant NSCLC [26]. Antiangiogenic agent was another candidate for the combination therapy. Promising outcomes were found in the Japanese JO25567 study, which evaluated the combination of erlotinib and bevacizumab [27]. The potential benefit will be further validated in the ongoing NEJ026 trial.

Other prognostic factors of distant metastasis included lymphovascular invasion and extranodal extension. The predictive effect of lymphovascular invasion on disease spread and early tumor recurrence has been demonstrated before [28,29]. It was associated with the pathologic type (adenocarcinoma) and the involvement of the N2 lymph node as well [30]. Extranodal extension also showed a remarkable impact on mortality of all causes. It occurred more frequently in the advanced tumor stage and in the adenocarcinoma subtype [31]. It is suggested that both of these pathologic features are carefully balanced in future studies assessing adjuvant systemic treatments.

Our study had several limitations. First, TKI was initially approved in China in 2005, but was not covered by medical insurance until 2017, which was responsible for the low administration rate in the early years, while chemotherapy had long been employed. The unbalanced follow-up time might cause bias. Besides, patients treated without adjuvant therapy usually had the association of lower economic class, old age, or physical intolerance, bringing potential confounding factors. Second, the pathologic data of some patients were missing because the pathologic specimens stored in the early years were not accessible. A well-designed randomized controlled trial is expected to validate the results.

## 5. Conclusions

In *EGFR*-mutant, completely resected stage III-pN2 lung adenocarcimona, adjuvant TKIs tended to improve distant control and disease-free survival compared with chemotherapy or the combination of both, with a favorable safety profile. This warrants investigation in randomized clinical trials to decide its potential on overall survival.

## Figures and Tables

**Table 1:** Patient characteristics.

Characteristics	No. (%)
	*N* = 274 (100)
Age	
≥60	116 (42.3)
<60	158 (57.7)
Sex	
Male	101 (36.9)
Female	173 (63.1)
KPS	
90–100	270 (98.5)
80	4 (1.5)
*EGFR* mutation	
Exon 19	145 (52.9)
Exon 21	129 (47.1)
Smoking	
Yes	74 (27.0)
No	200 (73.0)
N2 classification	
IIIA1-3	248 (90.5)
IIIA4	26 (9.5)
Surgery type	
Lobectomy	164 (94.3)
Pneumonectomy	5 (2.9)
Sublobectomy	5 (2.9)
Missing data	100 (/)
Visceral pleural invasion	
Yes	78 (29.4)
No	187 (70.6)
Missing data	9 (/)
Lymphovascular invasion	
Yes	90 (36.3)
No	158 (63.7)
Missing data	26 (/)
Perineural invasion	
Yes	12 (5.2)
No	219 (94.8)
Missing data	43 (/)
Extranodal extension	
Yes	80 (33.6)
No	158 (66.4)
Missing data	36 (/)
pT	
T1-2	246 (89.8)
T3-4	28 (10.2)
Adjuvant systemic therapy	
Adjuvant TKIs+chemotherapy	23 (8.4)
Adjuvant TKIs	52 (19.0)
Adjuvant chemotherapy	199 (72.6)
PORT	
Yes	23 (8.4)
No	251 (91.6)

*N*: number; KPS: Karnofsky Performance Score; *EGFR*: epidermal growth factor receptor; TKIs: tyrosine kinase inhibitors; PORT: postoperative radiation therapy.

**Table 2:** Adjuvant systemic treatments.

Regimen	No. (%)
Chemotherapy alone	199 (100)
Pemetrexed+cisplatin/carboplatin	165 (82.9)
Paclitaxel+carboplatin	7 (3.5)
Docetaxel+cisplatin/nedaplatin	7 (3.5)
Vinorelbine detartrate+cisplatin	20 (10.1)
TKIs alone	52 (100)
Gefitinib	20 (38.5)
Erlotinib	22 (42.3)
Afatinib	1 (1.9)
Icotinib	9 (17.3)
Combination treatment	23 (100)
Chemotherapy	
Pemetrexed+cisplatin/carboplatin	21 (91.3)
Paclitaxel+carboplatin	1 (4.3)
Docetaxel+cisplatin/nedaplatin	1 (4.3)
TKIs	
Gefitinib	12 (52.2)
Erlotinib	7 (30.4)
Icotinib	4 (17.4)

TKIs: tyrosine-kinase inhibitors.

**Table 3:** Univariable and multivariable analyses of prognostic factors on survivals.

Variable	DMFS			DFS			OS
	Univariable Analysis	Multivariable Analysis		Univariable Analysis	Multivariable Analysis		Univariable Analysis
	*p*	HR (95% CI)	*p*	*p*	HR (95% CI)	*p*	*p*
Age (≥60 vs. < 60)	0.130			0.182			0.786
Sex (male vs. female)	0.256			0.232			0.448
KPS (90–100 vs. 80)	0.719			0.958			0.425
*EGFR* mutation (exon 19 vs. 21)	0.134			0.025	0.90 (0.62–1.30)	0.576	<0.001
Smoking (yes vs. no)	0.505			0.522			0.374
N2 classification (IIIA1-3 vs. IIIA4)	0.586			0.912			0.257
Surgery type (lobectomy vs. pneumonectomy vs. sublobectomy)	0.520			0.526			0.198
Visceral pleural invasion (yes vs. no)	0.066	1.14 (0.70–1.85)	0.597	0.089	1.02 (0.61–1.71)	0.940	0.534
Lymphovascular invasion (yes vs. no)	<0.001	2.22 (1.38–3.57)	0.001	0.013	1.44 (0.95–2.18)	0.086	0.319
Perineural invasion (yes vs. no)	0.387			0.555			0.593
Extranodal extension (yes vs. no)	0.005	1.35 (0.85–2.14)	0.198	0.004	1.45 (0.96–2.19)	0.078	0.550
pT (T1-2 vs. T3-4)	0.390			0.723			0.410
Adjuvant systemic therapy	0.006		0.016	<0.001		0.002	0.413
TKIs vs. TKIs+chemotherapy	0.008	0.40 (0.17–0.94)	0.036	0.009	0.42 (0.19–0.94)	0.034	0.602
TKIs vs. chemotherapy	0.002	0.38 (0.20–0.73)	0.004	<0.001	0.33 (0.18–0.61)	<0.001	0.187
PORT (Yes vs. No)	0.288			0.356			

DMFS: distant metastasis-free survival; DFS: disease-free survival; OS: overall survival; HR: hazard ratio; CI: confidential interval; KPS: Karnofsky Performance Score; *EGFR*: epidermal growth factor receptor; TKIs: tyrosine kinase inhibitors; PORT: postoperative radiation therapy.

**Table 4:** Grade 3–4 treatment-related toxicities.

Grade 3–4 Toxicity	Chemotherapy Alone (*N* = 199)	TKIs Alone (*N* = 52)	Chemotherapy+TKIs (*N* = 23)
	No. (%)	No. (%)	No. (%)
Any Grade 3–4 toxicity	50 (25.1)	5 (9.6)	4 (17.4)
Anemia	9 (4.5)	1 (1.9)	0 (0)
Leukopenia	33 (16.6)	0 (0)	3 (13.0)
Neutropenia	20 (10.1)	0 (0)	1 (4.3)
Thrombocytopenia	13 (6.5)	0 (0)	1 (4.3)
Elevated ALT	2 (1.0)	1 (1.9)	1 (4.3)
Elevated AST	2 (1.0)	1 (1.9)	0 (0)
Rash	2 (1.0)	2 (3.8)	0 (0)
Diarrhea	2 (1.0)	1 (1.9)	1 (4.3)
Vomiting	8 (4.3)	0 (0)	0 (0)

TKIs: tyrosine kinase inhibitors; ALT: Alanine aminotransferase; AST: Aspartate aminotransferase.

## Supplementary Materials

The following are available online at https://www.mdpi.com/article/10.3390/curroncol28020135/s1, Table S1: Salvage treatments, Table S2: Patient characteristics (2015~present). Table S3: Univariable and multivariable analyses of prognostic factors on survivals (2015~present).
