# Supplementary material for: Benefit from Adjuvant TKIs Versus TKIs Plus Chemotherapy in EGFR-Mutant Stage III-pN2 Lung Adenocarcinoma
Source: Curr Oncol. 2021 Apr 7;28(2):1424–36. doi: 10.3390/curroncol28020135 (PMC8167779; doi:10.3390/curroncol28020135)
Supplement: Supplementary file 1 [file curroncol-28-00135-s001.pdf]

Supplementary Table I Salvage treatments

Treatment	N=149 (%)
TKIs alone	86 (57.7)
Radiotherapy alone	11 (7.4)
Chemotherapy alone	9 (6.0)
Chemotherapy+radiotherapy	4 (2.7)
Chemotherapy+TKIs	4 (2.7)
TKIs+surgery	2 (1.3)
TKIs+radiotherapy	2 (1.3)
TKIs+surgery+chemotherapy	2 (1.3)
Chemotherapy+surgery	2 (1.3)
Chemotherapy+radiotherapy+TKIs	2 (1.3)
TKIs+surgery+radiotherapy	1 (0.7)
Surgery+radiotherapy	1 (0.7)
Palliative treatment	14 (9.4)
Unknown	9 (6.0)

TKIs: tyrosine-kinase inhibitors.

Supplementary Table II Patient characteristics (2015~present)

Characteristics		No. (%) *N*=73 (100)
Age		
	⩾60	35 (47.9)
	<60	38 (52.1)
Sex		
	Male	27 (37.0)
	Female	46 (63.0)
KPS		
	90-100	72 (98.6)
	80	1 (1.4)
EGFR mutation		
	Exon 19	41 (56.2)
	Exon 21	32 (43.8)
Smoking		
	Yes	15 (20.5)
	No	58 (79.5)
N2 classification		
	IIIA1-3	69 (94.5)
	IIIA4	4 (5.5)
Surgery type		
	Lobectomy	58 (92.1)
	Pneumonectomy	4 (6.3)
	Sublobectomy	1 (1.6)
	Missing data	10 (/)
Visceral pleural invasion		
	Yes	24 (32.9)
	No	49 (67.1)
Lymphovascular invasion		
	Yes	45 (61.6)
	No	28 (38.4)
Perineural invasion		
	Yes	7 (10.3)
	No	61 (89.7)
	Missing data	5 (/)
Extranodal extension		
	Yes	43 (59.7)
	No	29 (40.3)
	Missing data	1 (/)
pT		

T1-2	69 (94.5)
T3-4	4 (5.5)
Adjuvant systemic therapy	
Adjuvant TKIs+chemotherapy	14 (19.2)
Adjuvant TKIs	16 (21.9)
Adjuvant chemotherapy	43 (58.9)
PORT	
Yes	4 (5.5)
No	69 (94.5)

KPS: Karnofsky Performance Score; *EGFR*: epidermal growth factor receptor; TKIs: tyrosine kinase inhibitors; PORT: postoperative radiation therapy.

Supplementary Table III Univariable and multivariable analyses of prognostic factors on survivals (2015~ present)

Variable	DMFS			DFS			OS
	Univariable analysis	Multivariable analysis		Univariable analysis	Multivariable analysis		Univariable analysis
	p	HR (95% CI)	*p*	*p*	HR (95% CI)	*p*	*p*
Age (⩾60 *vs* <60)	0.676			0.886			0.802
Sex (male *vs* female)	0.076	0.83 (0.38-1.81)	0.639	0.231			0.980
KPS (90-100 *vs* 80)	0.428			0.381			0.883
EGFR mutation (exon 19 *vs* 21)	0.046	0.41 (0.0031-53.71)	0.720	0.045	0.82 (0.40-1.70)	0.594	0.753
Smoking (yes *vs* no)	0.855			0.954			0.657
N2 classification (IIIA1-3 *vs* IIIA4)	0.297			0.179			0.574
Surgery type (lobectomy *vs* pneumonectomy *vs* sublobectomy)	0.692			0.592			0.936
Visceral pleural invasion (yes *vs* no)	0.683			0.833			0.318
Lymphovascular invasion (yes *vs* no)	0.018	3.66 (9.23-1.45)	0.006	0.143			0.791
Perineural invasion (yes *vs* no)	0.634			0.443			0.535
Extranodal extension (yes *vs* no)	0.216			0.259			0.220
pT (T1-2 *vs* T3-4)	0.166			0.321			0.116
Adjuvant systemic therapy	0.004		0.028	0.015		0.039	0.479
TKIs *vs* TKIs+chemotherapy	0.192	0.36 (0.085-1.52)	0.165	0.183	0.50 (0.14-1.77)	0.282	
TKIs *vs* chemotherapy	0.003	0.19 (0.054-0.67)	0.010	0.002	0.26 (0.087-0.78)	0.016	
PORT (Yes *vs* No)	<0.001	10.1 (40.04-2.55)	0.001	<0.001	6.37 (20.61-1.97)	0.002	

DMFS: distant metastasis-free survival; DFS: disease-free survival; OS: overall survival; HR: hazard ratio; CI: confidential interval; KPS: Karnofsky Performance Score; *EGFR*: epidermal growth factor receptor; TKIs: tyrosine kinase inhibitors; PORT: postoperative radiation therapy.
